# Is radiofrequency ablation of renal cancer metastases a suitable alternative to surgical metastectomy: A systematic review

**DOI:** 10.1007/s11845-025-04192-z

**Published:** 2025-11-28

**Authors:** Daniel Peter McNicholas, Eloise Dexter, Alexander Hampson, Jonathan Evans, Suresh Venugopal

**Affiliations:** 1 Department of Urology, Royal Liverpool University Hospital, Prescott street, Liverpool, Merseyside, L78XP United Kingdom; 2 Department of Interventional Radiology, Royal Liverpool University Hospital, Prescott street, Liverpool, Merseyside, L78XP United Kingdom

**Keywords:** Renal cell carcinoma, Kidney cancer, Metastatic RCC, Percutaneous ablation, Radiofrequency ablation, Metastectomy

## Abstract

**Background:**

Percutaneous ablative procedures such as radiofrequency ablation (RFA) are recognised as safe and effective treatment for small renal masses. These techniques are routinely used for metastases of other cancers such as lung or colorectal cancer.

**Aims:**

We aim to assess the feasibillity and safety of these treatments for metastatic RCC.

**Methods:**

A systematic review of the literature was performed searching for primary papers reporting outcomes on percutaneous ablative procedures such as RFA or cryoablation for the treatment of metastases of RCC. The study is registered with PROSPERO and is conducted in line with PRISMA guidelines.

**Results:**

Seven studies were identified with a total of 752 patients undergoing 961 radiofrequency ablation sessions for 1423 metastases were included in this systematic review. Of the seven studies, 4 were single centre retrospective cohort studies, 2 were multi-centre retrospective cohort studies and 1 was monocentric prospective study.

The mean age of patients in this study is 63.74 years and 34.4% were female. Mean follow up ranged from 10 to 61 months. Mean tumour size ranged from 12 to 79 mm. Overall Survival ranges from 50 to 100% from 5 years onwards post RFA. Overall we report a 30% risk of pneumothorax, 1.2% risk of non-pneumothorax complication and 0.1% risk of death per metastases treated.

**Conclusion:**

This is the first systematic review on this subject. We have shown that RFA can be safely used for metastatic RCC. It demonstrates acceptable oncological outcomes, comparable to surgical metastectomy.

## Introduction

Renal cell carcinoma (RCC) causes approximately 2% of all adult malignancies. With the increased numbers of abdominal imaging performed in recent years, the incidence of RCC diagnosis is on the rise [1, 2] Of all patients diagnosed with RCC, around a quarter have metastatic disease at the time of presentation. In those patients with only localised disease at presentation that are managed with radical treatment, approximately 50% will develop metastatic disease in the future. The TNM staging classification shows the 5-year survival for patients with metastatic RCC to be around 25% [1].

Treatment for metastatic RCC has evolved over recent years. It is a challenging disease to treat due to limited efficacy of chemotherapy treatment strategies, leading to poor outcomes for patients [1, 3] Systemic treatments such as immune checkpoint inhibitors as single agent or in combination with Vascular Endothelial Growth Factor (VEGF) targeted therapy have improved patient outcomes [3, 4]. However, they are associated with various adverse effects for the patient such as diarrhoea, rash, endocrinopathy and hepatic impairment [5]. In the oligometastatic setting patients may be offered metastectomy alone prior to undergoing radical nephrectomy. This can improve 5-year survival which ranges from 0 to 20% in the multple metastases setting, to 30–45% for those who undergo oligometastectomy [6–9]. Complete removal of all metastases has been shown to decrease the risk of death 2-fold [10]. In oligometastatic disease, the current European Association of Urology (EAU) guidelines recommend offering ablative therapies, including metastectomy, to facilitate local symptom control in patients with metastatic disease and favourable disease factors and in whom complete resection is possible [3]. Patients should undergo axial scanning to confirm disease status prior to metastectomy, to outrule rapidly progressive disease that may require systemic therapy [3].

Surgical metastectomy, radiotherapy and embolization are the main treatment modalities discussed for the management of oligometastases in RCC disease [3]. There is very limited evidence available in the literature regarding percutaneous ablative therapies such as RFA being offered as a treatment modality for metastases from RCC, despite it being offered as a treatment option for primary T1a renal tumours. For primary tumours, RFA has a technical success rate of > 95% and a ten-year disease-free survival up to 82% [3]. In this study, we are performing a systematic review of the literature to further evaluate the feasibility, safety and patient outcomes for RFA as a treatment modality for renal cell carcinoma metastases.

## Methods

### Search strategy

The review was prospectively registered (PROSPERO ID: 456123789) with a review question, search strategy, inclusion/exclusion criteria as detailed below. A systematic review was conducted in accordance with the Preferred Reporting items for Systematic Reviews and Meta-analyses (PRISMA) statement [11]. PubMed, Scopus and MEDLINE databases were searched for the period January 1989 to January 2025 inclusive for relevant publications.

#### Search terms

(metastatic OR metastases OR oligometastases) AND (“kidney cancer” OR “renal cancer”) AND (“percutaneous ablative therapy” OR “ablation therapy” OR “ablative therapy” OR cryotherapy).

### Review question

To assess the feasibility and safety of percutaneous ablative procedures such as RFA and cryoablation for the treatment of metastases of RCC.

### Eligibility criteria

No restrictions are being placed on the population included in the study.

Included:

Primary papers (including randomised control trials, prospective and retrospective case series, from both single centre or multiple centre studies) reporting outcomes for percutaneous ablative procedures such as RFA and cryoablation for the treatment of metastases of RCC.

Excluded:

- Papers published prior to 1989.
- Papers not in English language.
- Papers not reporting outcomes from RFA or cryoablation for renal cancer metastases.
- Case reports.
- Review articles.

Intervention:

RFA or cryoablation for the treatment of metastases of RCC.

### Data extraction

Two reviewers DM and ED independently reviewed the literature for primary studies looking at RFA or cryoablation for the treatment of metastases of RCC. Papers identified were reviewed and suitable papers meeting the inclusion criteria were included. Any disagreements between individual judgements were discussed with the study supervisor SV who is very experienced in academia and a consultant surgeon with > 10 years’ experience in RCC surgery.

The information was recorded on a Microsoft excel database.

The following data was be extracted and recorded:

- Primary studies reporting RFA or cryoablation for the treatment of metastases of RCC.
- Patient age and sex.
- Author names.
- Journal and year of publication.
- Total number of patients.
- Number of RCC metastases.
- Number of RFA or Cryoablation treatments performed.
- Tumour size.
- Nephrectomy status.
- Immunotherapy status.
- Time from nephrectomy to RFA or Cryoablation.
- Follow up duration.
- Overall survival.
- Complications.

### Outcome measures

#### Primary outcome

- Safety of Cryoablation or RFA for the treatment of metastases of RCC.

#### Secondary outcomes

- Overall survival.
- Follow up duration.
- Tumour size.

### Risk of bias assessment

The Newcastle Ottawa Scale (NOS) was used to assess the quality of the studies in this systematic review, with scores ranging from 0 to 9 points. The NOS is a review tool for evaluating risk of bias in observational studies [12]. The scale consists of four domains of risk of bias assessment; (i) selection bias; (ii) performance bias; (iii) detection bias and; (iv) information bias.

### Data reporting and statistical analysis

Data is presented as mean/median as described in text. Follow up and survival measures are in months. Meta-analysis is not performed due to small number of studies presenting meta-analysable data.

## Results

### Eligible studies

**Fig. 1:**
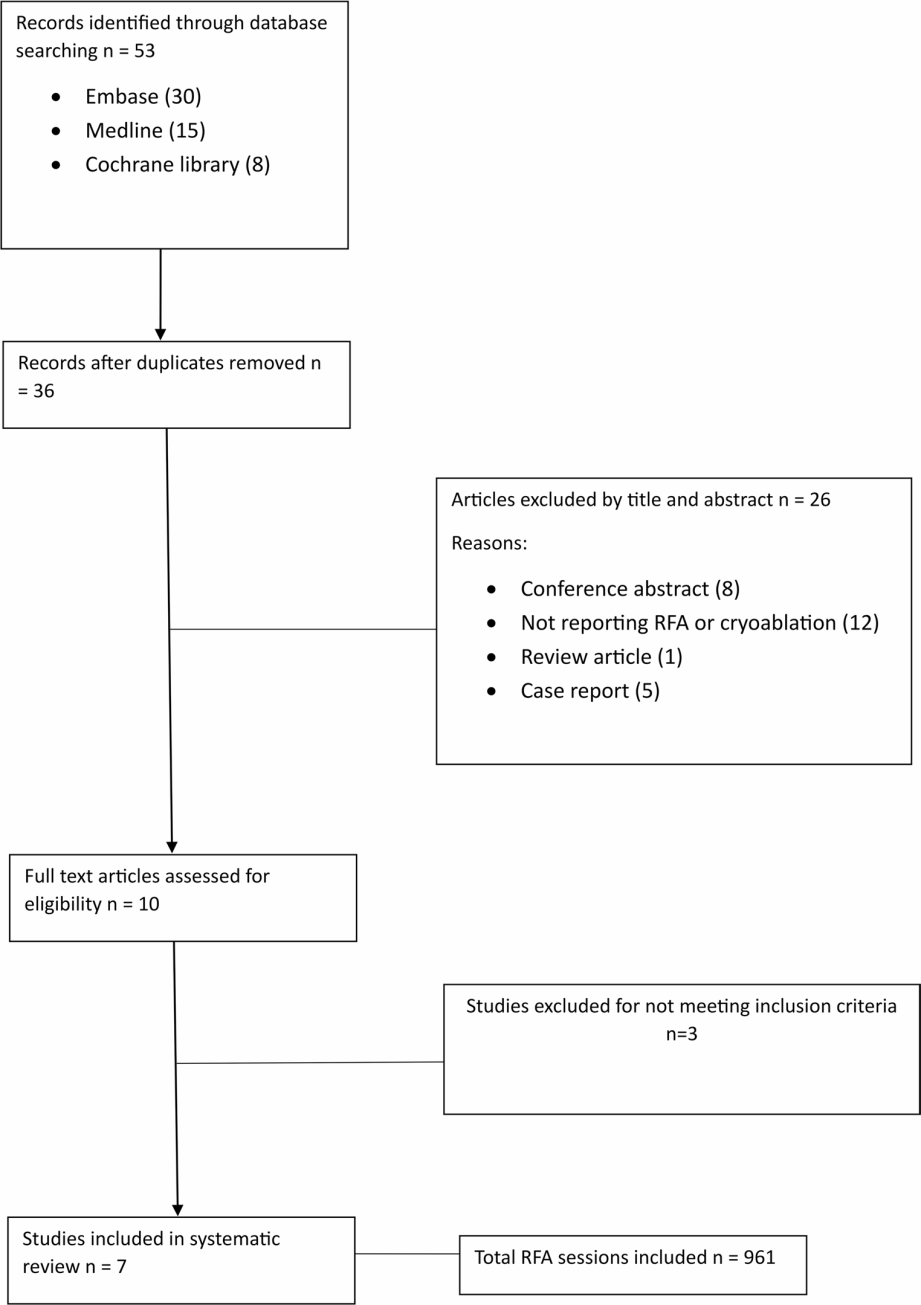
PRISMA (Preferred Reporting Items for Systematic Reviews and Meta-Analysis) diagram of the studies identified in the systematic review [11]

Seven studies were identified with a total of 752 patients undergoing 961 radiofrequency ablation sessions for 1423 metastases were included in this systematic review. Of the seven studies, 4 were single centre retrospective cohort studies, 2 were multi-centre retrospective cohort studies and 1 was monocentric prospective study. (Table 1). The initial search identified 53 articles and 10 full text articles were reviewed and assessed for eligibility, 7 of which were included (Fig. 1). All seven studies were in English and were all published between 2007 and 2021. The cohort of patients are reflective of modern practice, and all underwent RFA’s or metastases from renal cancer. The Newcastle Ottowa Scale was used to assess for risk of bias (Table 2) and study quality (Table 3) [12]. All seven studies included within the paper were classified as good quality, although there was a high risk of bias associated with all studies.

**Table 1:** Summary of study characteristics

Year Published	Country	Journal	Author Names	Study Type	Enrolment Dates	Total Patients	Number of metastases/RFA Sessions
2007	Germany	European Urology Supplements	Christian Kloeters et al.	Single centre retrospective cohort study	September 1999 to February 2007	38	66 metastases/66 sessions
2009	Australia	Annals of Surgical Oncology	Andrew Shu Yan Huo et al.	Single centre retrospective cohort study	August 2001 to June 2006	9	23 metastases/25 sessions
2009	Japan	British Journal of Urology Int.	Norihito Sogo et al.	Multi-centre retrospective cohort study	July 2001 to June 2008	39	135 metastases/89 sessions
2015	Japan	Radiology	Takaaki Hasegawa et al.	Single centre retrospective cohort study	February 2005 to May 2014	35	41 metastases/48 sessions
2015	France	Annals of Surgical Oncology	Thierry de Baère et al.	Multi-centre retrospective cohort study	December 2002 to December 2010	566	1037 metastases/642 sessions
2019	France	BioMed Central Cancer	Alexis Gonnet et al.	Single centre retrospective cohort study	Dates not provided. Collected over 11 years	53	100 metastases/65 sessions
2021	France	Cancers	Brice Chanez et al.	Single centre, prospective study	May 2017 to December 2019	12	21 metastases/26 sessions

**Table 2:** Newcastle-Ottawa scale risk of bias assessment adapted for Cohort Studies: A study can receive a maximum of one star for each numbered item in the Selection and Outcome categories. A maximum of two stars can be given for comparability [12]

**Study**	**S**				**C**	**O**			**Total**
	**Representativeness**	**Selection exposed to cohort**	**Ascertainment**	**Result not present at start of study**	**Comparability for confounders**	**Assessment of outcome**	**Follow up duration**	**Adequacy of follow up**	
Kloeters et al	*	-	*	*	*	*	*	-	6/9
Shu et al	*	-	*	*	*	*	*	*	7/9
Sogo et al	*	-	*	*	*	*	*	*	7/9
Hasegawa et al	*	-	*	*	*	*	*	*	7/9
Baère et al	*	-	*	*	*	*	*	*	7/9
Gonnet et al	*	-	*	*	*	*	*	*	7/9
Chanez et al	*	-	*	*	*	*	*	*	7/9

**Table 3:** Newcastle–Ottawa scale quality of evidence [12]. Good quality: 3 or 4 stars in selection domain AND 1 or 2 stars in comparability domain AND 2 or 3 stars in outcome/exposure domain. Fair quality: 2 stars in selection domain AND 1 or 2 stars in comparability domain AND 2 or 3 stars in outcome/exposure domain. Poor quality: 0 or 1 star in selection domain OR 0 stars in comparability domain OR 0 or 1 stars in outcome/exposure domain

Study	S				C	O			Total
	Representativeness	Selection exposed to cohort	Ascertainment	Result not present at start of study	Comparability for confounders	Assessment of outcome	Follow up duration	Adequacy of follow up	
Kloeters et al.	*	-	*	*	*	*	*	-	6/9
Shu et al.	*	-	*	*	*	*	*	*	7/9
Sogo et al.	*	-	*	*	*	*	*	*	7/9
Hasegawa et al.	*	-	*	*	*	*	*	*	7/9
Baère et al.	*	-	*	*	*	*	*	*	7/9
Gonnet et al.	*	-	*	*	*	*	*	*	7/9
Chanez et al.	*	-	*	*	*	*	*	*	7/9

#### Demographics

The mean age of patient in this study is 63.74 years. 34.4% of patients in the study were female. The full breakdown of demographics is included in Table 4.

**Table 4:** FA study characteristics

Study	Total Newcastle- Ottowa Score	Study quality
Kloeters et al. 2007	6	Good
Shu et al. 2009	7	Good
Sogo et al. 2009	**7**	**Good**
Hasegawa et al. 2015	**7**	**Good**
Baère et al. 2015	**7**	**Good**
Gonnet et al. 2019	**7**	**Good**
Chanez et al. 2021	**7**	**Good**

#### Oncological data

All studies reported metastatic renal cell carcinoma (mRCC). One paper by Hazegawa et al. reported RFA as a treatment modality for mRCC to the adrenals, it also included other tumour primary’s such as lung, colorectal and liver tumours [13]. Similarly de Baere et al. reported on metastases from RCC, colorectal, sarcoma and breast tumours that metastasised to the lungs [14]. Another paper by Chanez et al. specifically reported about mRCC to the pancreas only [15]. Soga et al., Gonnet et al., and Shu et al. all reported on RFA for mRCC to the lung [16–18]. Kloeters et al. reported on using RFA as treatment for RCC metastases to organs including lung, liver, kidney, adrenal and lymph nodes/soft tissue [19].

#### Number of metastases and treatments

Chanez et al. report 12 patients with 26 RFA procedures for 21 pancreatic metastases which were treated. 58% of cases were for one metastases, 17% of cases for 2 metastases and 5% of cases had 3–4 metastases. The mean treated tumour size was 1.7 cm. Other metastases included lung (*n* = 5), liver (*n* = 2), brain (*n* = 1), thyroid (*n* = 1), other (*n* = 2). All patients had a previous nephrectomy for RCC. The median interval between nephrectomy and RFA was 13.6 years. 33% were previously treated with immunotherapy.

Soga et al. report data on patients where RFA is with curative intent- when six or fewer lung metastases </= to 6 cm. They also report data where it is palliative in nature, when there were extra pulmonary lesions, seven or more lung lesions, or > 6 cm in size. All patients had previous nephrectomy.

Regarding the 15 patients for curative intent, 26 metastases were treated in total over 28 sessions. There were 8 single tumours and 7 patients had 2 tumours. The mean treated tumour size was 1.7 cm. All patients had a previous nephrectomy for RCC. The median interval between nephrectomy and RFA was 69.8 months. 66% were previously treated with immunotherapy. 7 patients had tumour recurrence.

Regarding the 24 palliative cases, 109 metastases were treated in total over 61 sessions. There was 1 patient with 1 tumour, 2 with 2 tumours, 1 with 4 tumours, 20 with > 7 tumours. The mean treated tumour size was 1.8 cm. All patients had a previous nephrectomy for RCC?. The median interval between nephrectomy and RFA was 46.7 months. 79% were previously treated with immunotherapy.

Shu et al. report data on 9 patients. There were 23 metastases treated with 25 sessions. The average tumour size was 1.84 cm. 44% of patients had previous immunotherapy. The mean size for ablation responsive tumours was 1.09 cm and the mean size for failed ablation tumours was 2.86 cm. 70% of tumours < 3 cm were ablated successfully. No ablations were successful > 3.5 cm.

Hasegawa et al. include a total of 35 patients, 9 with RCC related metastases. This paper specifically looked at RFA to adrenal metastases from RCC, as well as other tumour primaries. 35 patients underwent RFA for 41 metastatic adrenal tumours. 21 patients had tumours of < 3 cm and 20 for tumours ≥ 3 cm. 12 of the RFA procedures were combined with adrenal artery embolization.

Gonnet et al. report on 53 patients with a total of 100 metastases. 47% were single metastases, 21%, 17% and 15% had 2, 3 and 4–6 metastases respectively. 41 patients underwent 1 session of RFA and 12 had 2 sessions. 53% had neoadjuvant immunotherapy. The average tumour size was 1.2 cm. There were 9 tumour recurrences.

De Baere et al. recorded 68 patients with RCC. The paper reported outcomes of 566 patients with 1037 lung metastases including other primary cancers. Individual breakdown of number of RFA treatments and metastases for RCC was not given.

Kloeters et al. report 38 patients undergoing a total of 66 RFA’s. The intention was tumour volume reduction for 7 patients and for complete local tumour control in 31 patients. The ablated sites included liver (*n* = 39), lung (*n* = 11), kidney (*n* = 11), adrenal (*n* = 2) and lymph nodes or soft tissue (*n* = 3). All patients had previously had a nephrectomy. 7 patients had immunotherapy within 3 months prior to RFA.

Those treated with intention of volume reduction were due to tumour volume being > 5 cm in size on CT imaging. The average tumour size was 7.9 cm. There 16 metastases treated in total. 2 patients had one lesion, 4 patients had 2 lesions, and 2 patients had 3 lesions.

31 patients were treated with the intention of complete tumour ablation. There were 50 metastases in total, with a mean size of 23 mm and ranging from 10 to 50 mm. 27 patients had a solitary tumour, 3 had 2 lesions and one had 3 lesions.

#### Survival outcome, progression and follow up

Channez et al. reports a median follow up duration of 27.7 months (6.4–57.1 months). Of the 21 pancreatic metastases treated, 40% had complete response at 12months. Focal control was achieved in 73.3% at 12 months. Progressive disease was observed in 26.7% at 12 months. Median progression free survival was 25.38 months.

Sogo et al. had a mean follow up of 25 months. Local tumour progression was found in 13 of the 135 treated tumours (9%) and one third of patients (13/39). Maximum tumour diameter was a significant factor affecting local tumour progression. It was 7% in 120 tumours < 3 cm and 33% in those > 3 cm. The curative cohort were followed up for 24.8 months (1.3–69.5) and the palliative cohort were followed up 28.9 (1–69.8.8 months). The overall survival rate for the curative group was 100% at 5 years, however in the palliative group it was 90% and 52% at 1 and 5 years respectively. In the curative group, 40% of patients had recurrences in the lung after RFA treatment.

Shu et al. followed up patients for a mean length of 19.4 months. Median post RFA survival was 21 months. 36% increased in size (no time frame given).

Hasegawa et al. had mean follow up of 30 months. 4 of the 9 patients had extra adrenal metastases. Some of these received RFA to extra adrenal metastases and others received systemic therapy, but the breakdown is not given. No patient with extra adrenal metastases at the time of adrenal RFA achieved disease free status. The 3-year survival recorded was 33% and 100% for those with extra adrenal metastases and localised adrenal metastases respectively.

Gonnet’s paper had a mean follow up of 61 months. 9 patients had local recurrence. 1 and 5 year survival was 94 and 61.8% respectively.

De Baere et al. had a mean follow up of 35.5 months. Treatment failure for RFA for RCC was 7.4%, 13% and 25.1% at 1, 2 and 3 years respectively. Overall survival was 95.5%, 73.5% and 53.8% at 1, 2 and 3 years respectively. Progression free survival was 39.7%, 13.8% and 9.2% at 1, 2 and 3 years respectively.

Kloeters et al. describes a mean of 10 months (3–47) follow up. There were no deaths in the observation period.

#### Complications

Chanez et al. report all patients spent 2 days in hospital. 2 patients had CD IIIb complications. On developed a duodenal abscess, the second developed a hepatic abscess. Both were the only patients continuing with TKI’s treatment at time of their RFA.

Soga et al. report pneumothorax (PTX) requiring chest drain was the most common complication, occurring in 7% of cases. Aspiration pneumonia happened in one case. Minor complications such as minor pneumothorax, pleural effusion and haemosputum occurred < 5%.

Shu et al. report 5 pneumothoraces, of which 3 treated with chest drains. Patients with PTX had average of 3 tumours ablated whereas those without had 1 ablated. One patient had 9 ablations and developed a bronchopulmonary fistula, pleural effusion and PTX. One patient developed pneumonia.

Hasegawa et al. reported 1 case of Acute renal failure and Adrenal failure, 1 case of heart failure, 1 case of a stroke with hypertensive crisis. 1 case of a Hepatic subcapsular hematoma with hypertensive crisis. There were 17 cases of a hypertensive crisis, 1 case of a hypertensive crisis with adrenal failure, 1 case of hypertensive crisis with heamothorax, 1 case of hypertensive crisis with pneumothorax, and a case of adrenal failure.

Gonnet et al. paper- 39 pneumothorax (26 drained), 32 pleural effusions, 4 pneumonia.

De Baere et al. reported that two patients died within the 30 days postoperative period. One from decompensated cardiorespiratory function and the other from cerebral stroke. Additionally, pneumothoraces were seen in in 67% of the procedures, of those 28% needed no treatment, 14% required simple aspiration during the RFA procedure and 58% required a chest tube. Two haemothoraces related to intercostal artery puncture were treated with embolization during the same procedure and four minor skin burns occurred.

Kloeters et al. describe 2 pneumothoraces, managed conservatively.

## Discussion

Since the first description of image guided ablation in 1989 for liver cancer, the treatment has evolved to treat a variety of malignancies [20]. Nowadays, RFA has been shown to be a safe, effective and nephron sparing treatment for renal cell carcinoma, with low rates of recurrence and low morbidity. It’s 5-year cancer specific survival is 97% and it has a low risk of major complications (5%) [21]. It is a very good alternative to robotic assisted partial nephrectomy for smaller renal tumours < 3 cm due to its nephron sparing effect and minimally invasive nature [21]. However, it’s use to treat metastases from RCC is not well documented in the literature or mentioned in major urological guidelines. It is well known that RFA is effective at treating other cancers in organs such as liver, lung and bone [22–24], all of which are common sites for renal cancer metastases, 20%, 45% and 29% respectively [25]. The effectiveness of RFA for renal tumour pathology is evident, as is the feasibility of accessing metastases in the most common site of renal tumour metastases in the treatment of other cancers. In this review, we have described over 1400 RCC metastasis to locations including lung, liver, pancreas, adrenal, brain and thyroid. None of the authors describe any difficulty with access to the respective tumours. Therefor feasibility of RFA for treatment of RCC metastases is evidently clear. However, other treatment modalities such as surgical metastectomy, radiotherapy and embolization are more popular.

Metastectomy can offer a significant survival benefit. A paper by Wu et al. in 2019 of almost 3000 metastatic RCC patients showed metastectomy was associated with a decreased cancer specific mortality [26]. Many papers have shown metastectomy is associated with improved overall survival when compared with those not undergoing metastectomy treatment [27, 28.] A systematic review has shown that surgical metastectomy has been associated with median overall survival ranging from 36 to 142 months for those undergoing complete resection, compared to 8–27 months when the resection is incomplete [27]. In our review there was heterogeneity in the reporting of outcomes from the various papers, however the outcomes were largely encouraging. Chanez et al. reported a progression free survival (PFS) of 25.38 months [15]. Shu et al. reported a post RFA survivaL of 19.4 months and Kloeters et al. reported no deaths in the first 10 months post RFA [1, 16]. Overall survival (OS) was reported by Gonnet et al. as 61.8% at 5 years, and by Hasegawa et al. as 100% at 3 years for patients with localised adrenal metastasis, otherwise 33% at 3 years for extra-adrenal metastases [2, 13]. The largest study in our review, conducted by De Baer et al. and involving over 550 patients, reported a 3-year overall survival (OS) rate of 53%, a 5-year OS rate of 51%, and a median OS of 62 months [14]. These results align with contemporary data on surgical lung metastectomy for colorectal malignancies [14]. Soga et al. had a 100% 5 year OS in those treated with curative intent, decreasing to 50% for those treated palliatively [25]. Therefor we can see OS ranges from 50 to 100% from 5 years onwards post RFA. This is similar to the outcomes described earlier from surgical metastectomy. Interestingly, both Hasegawa et al. and Soga et al. focused exclusively on patients with metastases that were considered surgically unresectable in their studies [13, 25]. This highlights that RFA is an alternative option to surgery, whether surgery is feasible or not.

Another alternative treatment option to both surgical metastectomy and RFA is stereotactic ablative body radiotherapy (SABR). Traditionally, kidney tumours have not been treated with radiotherapy. This is because RCC is thought to be relatively radio-resistant [28]. However, there has been an increasing body of evidence in recent years regarding SABR as a treatment option for kidney cancer. The FASTRACK II trial recently published in The Lancet has shown that SABR is a safe and effective treatment for renal cancer and has paved the way for further trials in this area [29]. More specifically to RCC metastases, a review by Ali et al. which was published in European Urology has reviewed the literature looking at SABR as a management option for primary and metastatic RCC. They found it had effective local control in 90% of patients with intra-cranial metastases, and it was able to delay systemic therapy for at least 1 year in oligometastatic RCC, with low risk of toxicity. They have shown it is safe, feasible and tolerable as a treatment approach [30]. While these results are promising, this treatment is not yet offered outside of clinical trials.

It is important to consider the safety profile of RFA for treatment of metastases, in comparison to surgery which is the current most common treatment modality used. Understandably, the risks of surgery vary greatly dependant on the organ being operated on. In this systematic review, over 670 patients and more than 1,150 metastases underwent RFA for lung metastases. Pneumothorax was the most prevalent complication, affecting 431 patients in our review. De Baere, Gonnet and Shu all performed RFA only on lung tumours and all reported > 50% pneumothorax rates [1, 2, 14]. In de Baer’s paper, 67% of patients required chest drain and 2 days admission. It was highlighted in de Baer’s paper that RFA is well tolerated and spares the lung parenchyma, causing no change to respiratory function, even after multiple treatments [14]. Other than pneumothorax, there were 17 reported complications and 2 deaths. Only one paper reported complications using the Clavien-Dindo classification [15]. Hasegawa et al. and Gonnet et al. reported 8% and 3% major complication rate respectively [2, 13]. Overall we report a 30% risk of pneumothorax, 1.2% risk of non-pneumothorax complication and 0.1% risk of death per metastases treated. This is significantly less than those complications reported in a large population based assessment by Meyer et al. Including over 1000 surgical metastectomy’s for mRCC patients. Their complication rate was 45.7%, with major complications (CD III-IV) comprising 27.5% [31]. These results highlight that RFA is as safe as surgery for the treatment of RCC metastases.

The landscape for management of metastatic RCC is evolving in recent years. As previously mentioned, the use of SABR may have a role to play in the future, and there are also continued improvements being made regarding novel immune checkpoint inhibitors (ICI) in the treatment of this disease, which is now standard of care [32–37]. Patients have been successfully treated with ICI with/without oligometastectomy, resulting in the disease being downstaged, and they are subsequently treated by radical nephrectomy with curative intent. Patients treated with oligometastectomy are high risk for recurrence, and metachronous metastases in the first year are associated with poor prognosis [32]. The Keynote 564 trial found that pembroluzimab treatment led to significant improvement in disease free survival after surgery in high-risk patients, including those who underwent oligometastectomy [32]. Given the increasing number of patients that will be suitable for oligometastectomy in the future as a result of this, it is important to consider RFA as a suitable alternative to surgery. Furthermore, Chanez paper highlights that the activation of the immune system by performing RFA in association with ICI treatment may lead to an even more efficient tumour response [15].

To the best of the authors knowledge we have performed the first systematic review of the literature on this topic. There is a lack of research in this area and we hope that this paper will highlight the option of RFA as a safe and effective alternative to surgery in the multi-modal treatment and potential cure of patients with metastatic RCC. With the new ICI drugs which can downstage disease, aswell as treating patients with oligometastases with curative intent, the treatment of metastatic RCC is rapidly evolving. RFA is an underutilised and particularly useful alternative treatment for metastases, where surgery would be considered very high risk due to tumour location, where the tumour is surgically unresectable or in the co-morbid patient. More studies with larger patient numbers and more homogenous data recording are required to give more robust data on this subject. In the current climate where SABR is being investigated for suitability in management of this disease, a trial comparing surgical metastectomy, SABR and RFA would be helpful to fully quantify the oncological outcomes between the 3 treatments and compare the safety profile more closely.
